# Frequency Domain Specifications Based Robust Decentralized PI/PID Control Algorithm for Benchmark Variable-Area Coupled Tank Systems

**DOI:** 10.3390/s22239165

**Published:** 2022-11-25

**Authors:** Achu Govind K.R., Subhasish Mahapatra

**Affiliations:** School of Electronics Engineering, VIT-AP University, Amaravati 522237, Andhra Pradesh, India

**Keywords:** decouplers, TITO system, coupled tank systems, FOPDT model, model uncertainty, robust control

## Abstract

A decentralized PI/PID controller based on the frequency domain analysis for two input two output (TITO) coupled tank systems is exploited in this paper. The fundamentals of the gain margin and phase margin are used to design the proposed PI/PID controller. The basic objective is to keep the tank at the predetermined level. To satisfy the design specifications, the control algorithm is implemented for decoupled subsystems by employing a decoupler. First-order plus dead time (FOPDT) models are obtained for the decoupled subsystems using the model-reduction technique. In addition, the control law is realized by considering the frequency domain analysis. Further, the robustness of the controller is verified by considering multiplicative input and output uncertainties. The proposed method is briefly contrasted with existing techniques. It is envisaged that the proposed control algorithm exhibits better servo and regulatory responses compared to the existing techniques.

## 1. Introduction

The automatic regulation of liquid level is an essential factor in most of the process control industries, such as food processing, water purification, the chemical reactors and pharmaceutical industry. Maintaining the desired level in a coupled tank system is one of the challenging problems due to the non-linear behavior of the multi-input multi-output (MIMO) systems. An essential characteristic of MIMO systems is cross-coupling and interaction between the variables, which lead to poor loop performance. Further, the time delay and uncertainties affect the closed-loop performance. Hence, controlling the parameters in presence of interaction is very complex relative to single-loop systems. The following articles were explored for various control strategies for interacting tank systems.

Conventional PID controllers are widely used in various fields, such as image processing, process industries as exploited in [1,2,3] because of their structural simplicity, design easiness, availability of various tuning methods, etc. However, the system performance is affected in the presence of non-linearities and system uncertainties. An adaptive fuzzy logic controller with the Kalman algorithm for regulating the level of conical tank system is reported in [4]. The fuzzy rules are defined based on the Kalman filter algorithm. Further, the controller parameters are adjusted based on the defined algorithm to attain the desired servo response. A comparative analysis is carried out between the adaptive passivity-based controller (APBC) and fractional-order APBC (FOAPBC) as discussed in [5]. However, it is inferred that FOAPBC is able to reduce the overshoot by attaining the desired response. As described in [6], a reinforcement learning is used for designing a controller to regulate the level of a conical tank system. The learning algorithm is based on the Q-learning technique. A fractional-order proportional integral derivative controller (FOPID) for a conical tank system is addressed in [7]. An objective function is defined to reduce the error between the plant model and the reference model. Further, FOPID controller parameters are determined by particle swarm optimization. Various optimization techniques for regulating the level of conical tank systems are presented in [8]. However, it is found that the required design criteria can be attained with the bubble net whale optimization algorithm. A fuzzy-based sliding mode controller for regulating the level of a spherical tank system is reported in [9]. The system is able to satisfy the design specifications. In [10], a PI controller is designed based on the root locus method for regulating the level of the spherical tank system. Various multi-model control techniques for maintaining the level of spherical tank systems are described in [11,12,13,14,15]. The tuning of PI controllers with a genetic algorithm for regulating the level of spherical tank system is addressed in [16]. The stability analysis of the quadruple tank system with multi-variable dead time is exploited in [17]. Further, the controller can attain the desired design specifications by exhibiting the closed loop stability. In [18], a hybrid controller comprising of a sliding mode technique and a state feedback algorithm for a quadruple tank system is presented. The transient response is guaranteed by the sliding mode controller, while the state feedback algorithm reduces the steady-state error. A multi-level switching fractional-order sliding mode controller satisfying the servo and regulatory responses for a quadruple tank system is reported in [19]. A robust PI controller ensuring the servo response for a quadruple tank system is discussed in [20]. A model predictive controller based on linear quadratic Gaussian regulator for achieving the design criteria of the quadruple tank system is described in [21]. A multivariable controller is designed based on the equivalent loop transfer function in [22]. The controller was able to reduce the loop interactions through iterative procedure. Further, the robustness of the controller is verified with the Nyquist plot analysis. Hence, it can be inferred from the aforementioned literature that the loop interactions affect the performance of the system in centralized structures. By designing off-diagonal controllers or full controller structures, the loop interactions can be reduced. However, the design process becomes complex as the individual loop controllers need to be tuned independently. Hence, decentralized controllers are preferred due to their simple structure, as tuning is required only for diagonal systems. Some of the recent developments in the decentralized scheme are discussed below.

An adaptive decentralized control technique for a coupled tank system is addressed in [23]. In this, the PI controller can satisfy the desired servo response. Further, stability of the system is analyzed with multi-variable Nyquist plot. A dynamic matrix controller for a coupled conical tank system is exploited in [24]. The controller is able to satisfy the design criteria for the entire operating range. Although the servo response is attained, the controller fails to satisfy the regulatory response. In [25], a fractional order PI/PID controller for an interacting conical tank system is presented. The controller is designed based on the equivalent transfer function model and simplified decoupler. Further, the bat optimization algorithm is used to obtain the controller parameters. A fuzzy fractional-order controller for a coupled conical tank system is reported in [26]. The controller parameters are optimized through a metaheuristic algorithm. Further, the stability is analyzed with the Lyapunov theorem. In [27,28,29], PI/PID are designed to control the level of spherical tank systems using gain scheduling and fractional-order fuzzy algorithms. Further, comparative analysis is made with other controllers based on the performance indices. In [29], decouplers are designed based on the inverted decoupling scheme. A multi-model cascade control structure is exploited in [30] to regulate the level of the coupled spherical tank system. Various model predictive controllers for quadruple tank systems are presented in [31]. However, it is inferred that the multi-parametric model decentralized PI controller is able to satisfy the servo and regulatory responses. In [32], a relay-based PID tuning technique is reported for the quadruple tank system. Similarly, a sliding mode controller for a quadruple tank system is discussed in [33]. The process delays are compensated with the Pade approximation technique. Further, the stability is verified with the Lyapunov theorem. A higher-order sliding mode controller for the quadruple tank system is described in [34]. A nonlinear disturbance observer is addressed in [35] for the quadruple tank system. The controller is designed based on coupling characteristics. In [36], a hybrid controller for maintaining the water level of the coupled tank system is reported. However, there are some decentralized control schemes, where the use of decouplers is not essential. A decentralized PID controller where the coupling effects are reduced is discussed in [37]. The controller parameters are derived from the optimization problem which is defined based on stability, robustness and performance criteria. An optimal PID tuning design procedure is presented in [38] by ensuring the closed loop stability. Further, the controller parameters are derived from the optimization of the stability margin. Although a justifiable closed-loop response is achieved with various controllers as reported in the aforementioned literature, there exists a trade-off between the robustness and controller performance. This work focuses on designing a decentralized PI/PID controller for maintaining the level of interacting coupled tank systems. Decouplers are designed to reduce the loop interactions. Further, the decentralized PI/PID controller is designed based on the specifications of gain and phase margins. The proposed controller is intended to attain the desired set point, irrespective of the disturbances and parameter uncertainties. The robust behavior of the system is verified in the presence of disturbances and uncertainties. The main contribution of this paper are as follows:

- Design of the reduced order FOPDT model for the coupled tank systems using frequency domain specifications.
- Design of a decentralized PI/PID controller for achieving both servo and regulatory responses.
- Analysis of the efficacious behavior of the developed control algorithm by comparing the proposed technique with existing methods.
- Verification of the robust behavior in presence of model uncertainties.

The paper is organized as follows: The problem statement is described in Section 2. Section 2.1 deals with the decoupled system design followed by the controller design in Section 3. Simulation results are discussed in Section 4. The conclusion is discussed in Section 5.

## 2. Problem Statement

This section describes the modeling of decouplers for two input two output (TITO) systems. The problem statement is formulated in order to control the level of various coupled tank systems. The generalized representation for a TITO system is given by Equation (1):

(1) $$G\left(s\right)=\left[\begin{array}{cc} g_{11}\left(s\right)e^{-\mu_{11}s} & g_{12}\left(s\right)e^{-\mu_{12}s}\\ g_{21}\left(s\right)e^{-\mu_{21}s} & g_{22}\left(s\right)e^{-\mu_{22}s} \end{array}\right]$$

The decouplers are designed to reduce the control loop interactions. Here, the controller output is the decoupler input while the decoupler output is the process input. The control technique is realized to reduce the loop interactions to obtain the desired response.

### 2.1. Decoupled System Design

**Figure 1:**
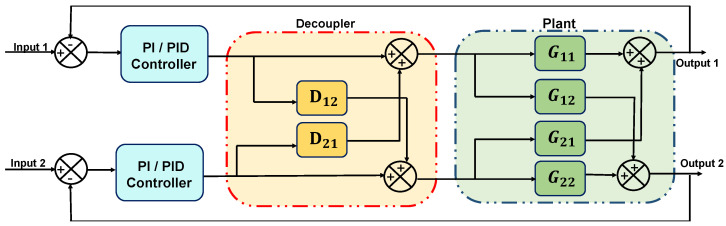
Closed loop decoupled structure for a TITO system.

This subsection exploits the adopted decoupling strategy. As reported in [39], under an inverted decoupling scheme, the manipulated input in one loop is determined by computing the weighted sum of its own controller output and the manipulated output from the other loop. The general block diagram of the TITO system is illustrated in Figure 1.

The decoupler matrix for a TITO system is given by

(2) $$D\left(s\right)=\left[\begin{array}{cc} 0 & -\frac{g_{12}\left(s\right)}{g_{11}\left(s\right)}\\ -\frac{g_{21}\left(s\right)}{g_{22}\left(s\right)} & 0 \end{array}\right]$$

Thus, the transfer matrix of the process is given as H(s) is given by

(3) $$H\left(s\right)=G\left(s\right)*D\left(s\right)=diag\{h_{11}\left(s\right),h_{22}\left(s\right)\}$$

By the proposed controller the decoupled elements $h_{jj}$ needs to be controlled where j=1,2.

### 2.2. Reduced Order FOPDT Model

Due to the complex dynamics of the diagonal elements in Equation (3), the process of designing the decoupling controller is difficult. Hence, model reduction techniques need to be applied to obtain the FOPDT model. The process dynamics can be approximated with the FOPDT model. The reduced model can be approximated as

(4) $$G_{jj}\left(s\right)=\frac{K_{jj}e^{-\Phi_{jj}s}}{T_{jj}s+1},j=1,2$$

The frequency response fitting is obtained at two points Ω = 0 and $\Omega =\Omega_{Cjj}$ to determine the unknown:

(5) $$\begin{array}{ccc} G_{jj}\left(0\right) & = & h_{jj}\left(0\right) \end{array}$$

(6) $$\begin{array}{ccc} |G_{jj}\left(j\Omega_{Cjj}\right)| & = & |h_{jj}\left(j\Omega_{Cjj}\right)| \end{array}$$

(7) $$\begin{array}{ccc} ∠\left\{G_{jj}\left(j\Omega_{Cjj}\right)\right\} & = & ∠\left\{h_{jj}\left(j\Omega_{Cjj}\right)\right\} \end{array}$$

The FOPDT parameters is determined as

(8) $$\begin{array}{ccc} K_{jj} & = & h_{jj}\left(0\right) \end{array}$$

(9) $$\begin{array}{ccc} T_{jj} & = & \sqrt{\frac{K_{jj}^{2}-{\left|h_{jj}\left(j\Omega_{Cjj}\right)\right|}^{2}}{|h_{jj}\left(j\Omega_{Cjj}\right){|}^{2}\Omega_{Cjj}^{2}}} \end{array}$$

(10) $$\begin{array}{ccc} \Phi_{jj} & = & \frac{\pi +tan^{-1}\left(-\Omega_{Cjj}T_{jj}\right)}{\Omega_{Cjj}T_{jj}} \end{array}$$

## 3. Controller Design

The design of the decentralized PI/PID controller is explored in this section. The gain and phase margin (GPM) are linked with the frequency response of the system. The robustness of the system can be analyzed with the help of GPM.

### 3.1. Design of PI Controller

**Figure 2:**
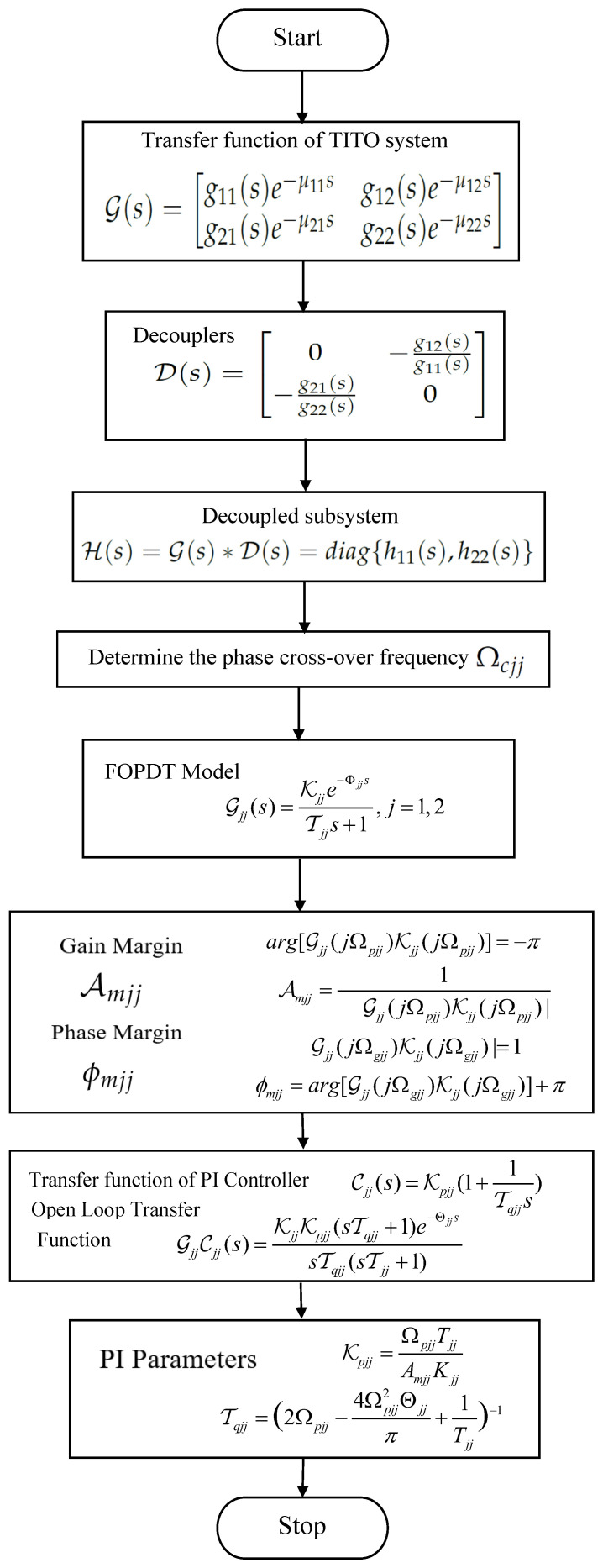
Flow chart for PI controller design.

The flow chart for the PI controller design is shown in Figure 2. The phase and amplitude equations can be derived from the fundamentals of GPM as

(11) $$\begin{array}{c} arg[G_{jj}\left(j\omega_{Pjj}\right)K_{jj}\left(j\omega_{Pjj}\right)]=-\pi \end{array}$$

(12) $$\begin{array}{c} A_{Mjj}=\frac{1}{|G_{jj}\left(j\omega_{Pjj}\right)K_{jj}\left(j\omega_{Pjj}\right)|} \end{array}$$

(13) $$\begin{array}{c} |G_{jj}\left(j\omega_{Gjj}\right)K_{jj}\left(j\omega_{Gjj}\right)|=1 \end{array}$$

(14) $$\begin{array}{c} \phi_{mjj}=arg\left[G_{jj}\left(j\omega_{Gjj}\right)K_{jj}\left(j\omega_{Gjj}\right)\right]+\pi , \end{array}$$

The PI controller is given as

(15) $$C_{jj}\left(s\right)=K_{Pjj}\left(1+\frac{1}{T_{Qjj}s}\right)$$

The open loop transfer function is derived from Equations (4) and (15) as

(16) $$G_{jj}C_{jj}\left(s\right)=\frac{K_{jj}K_{Pjj}\left(sT_{Qjj}+1\right)e^{-\Phi_{jj}s}}{sT_{Qjj}\left(sT_{jj}+1\right)}$$

Due to the arctan function, the PID controller parameters are derived numerically for the desired GPM. The arctan function is approximated to obtain an analytical solution and is given as

(17) $$\arctan x=\left\{\begin{array}{cc} \frac{1}{4}x, & \left(\left|x\right|\leq 1\right)\\ \frac{1}{2}\pi -\frac{\pi }{4x}, & \left(\left|x\right|>1\right) \end{array}\right.$$

where *x* can be either of $\omega_{Pjj}T_{jj}$, $\omega_{p}T_{Qjj}$, $\omega_{G}T_{jj}$, $\omega_{G}T_{Qjj}$. The resulting PI controller parameters are

(18) $$K_{Pjj}=\frac{\omega_{Pjj}T_{jj}}{A_{mjj}K_{jj}}$$

(19) $$T_{Qjj}={\left(2\omega_{Pjj}-\frac{4\omega_{Pjj}^{2}\Phi_{jj}}{\pi }+\frac{1}{T_{jj}}\right)}^{-1}$$

where

(20) $$\omega_{Pjj}=\frac{A_{Mjj}\phi_{mjj}+\frac{1}{2}\pi \left(A_{Mjj}-1\right)}{\left(A_{Mjj}^{2}-1\right)\Phi_{jj}}$$

### 3.2. Design of PID Controller

**Figure 3:**
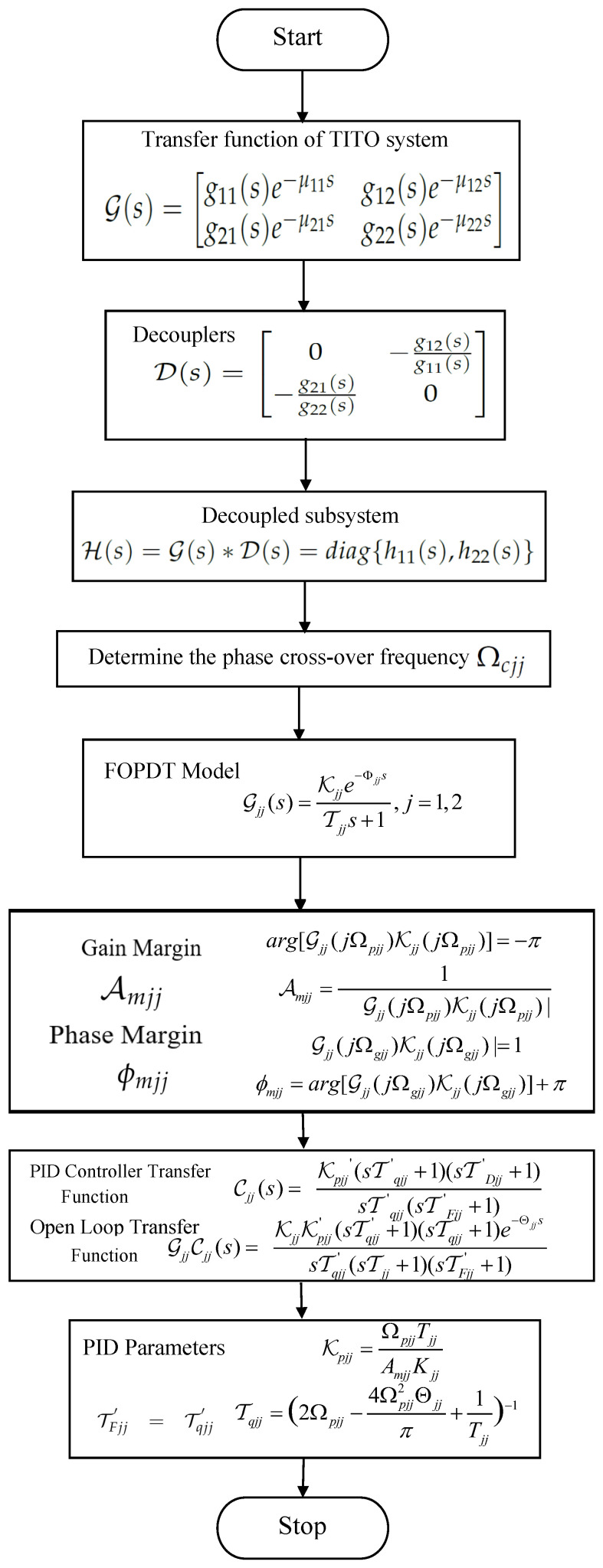
Flow chart for PID controller design.

The flow chart for the PID controller design is shown in Figure 3.

Similarly, the PID controller in series form can be expressed as

(21) $$C_{jj}\left(s\right)=\frac{K_{Pjj}^{{}^{\prime }}\left(sT_{Qjj}^{{}^{\prime }}+1\right)\left(sT_{Djj}^{{}^{\prime }}+1\right)}{sT_{Qjj}^{{}^{\prime }}\left(sT_{Fjj}^{{}^{\prime }}+1\right)}$$

The derivative time $T_{Dii}$ for the PID controller is typically chosen as a constant ratio of the integral time. Hence,

(22) $$T_{Djj}^{{}^{\prime }}=T_{Qjj}^{{}^{\prime }}$$

Hence, the open loop transfer function is derived from the Equations (4), (21) and (22)

(23) $$G_{jj}C_{jj}\left(s\right)=\frac{K_{jj}K_{Pjj}^{{}^{\prime }}\left(sT_{Qjj}^{{}^{\prime }}+1\right)\left(sT_{Qjj}^{{}^{\prime }}+1\right)e^{-\Phi_{jj}s}}{sT_{Qjj}^{{}^{\prime }}\left(sT_{jj}+1\right)\left(sT_{Fjj}^{{}^{\prime }}+1\right)}$$

By considering $T_{Fjj}^{{}^{\prime }}$ = $T_{jj}^{{}^{\prime }}$, Equation (23) can be simplified as

(24) $$G_{jj}C_{jj}\left(s\right)=\frac{K_{jj}K_{Pjj}^{{}^{\prime }}\left(sT_{Qjj}^{{}^{\prime }}+1\right)e^{-\Phi_{jj}s}}{sT_{Qjj}^{{}^{\prime }}\left(sT_{jj}+1\right)}$$

The resulting PID controller parameters are

(25) $$\begin{array}{ccc} K_{pjj}^{{}^{\prime }} & = & \frac{\omega_{Pjj}T_{jj}}{A_{Mjj}K_{jj}} \end{array}$$

(26) $$\begin{array}{ccc} T_{qjj}^{{}^{\prime }} & = & {\left(2\omega_{Pjj}-\frac{4\omega_{Pjj}^{2}\Phi_{jj}}{\pi }+\frac{1}{T_{jj}}\right)}^{-1} \end{array}$$

(27) $$\begin{array}{ccc} T_{Fjj}^{{}^{\prime }} & = & T_{Qjj}^{{}^{\prime }} \end{array}$$

where $\omega_{Pjj}$ is defined in Equation (20).

### 3.3. Analysis of Robustness

**Figure 4:**
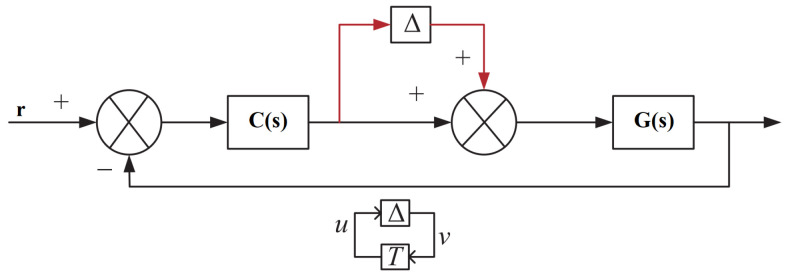
Multiplicative input uncertainty schematic structure.

**Figure 5:**
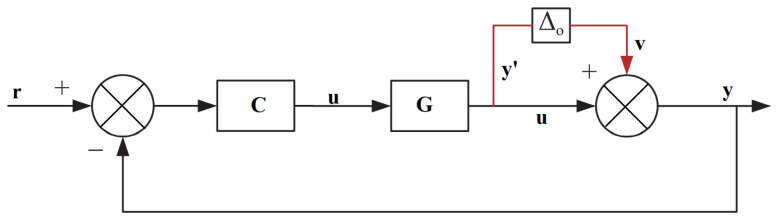
Multiplicative output uncertainty schematic structure.

Due to the unmodeled process dynamics which occurs in real time, the robust analysis of the proposed controller is essential in the closed-loop control system. There are various sources of uncertainty that can affect system performance which in turn affects the stability of the system. Therefore, both multiplicative input and multiplicative output uncertainties are combined into the model to examine the stability of the developed system. The schematic arrangement of multiplicative input and multiplicative output uncertainty in T−Δ form is shown in Figure 4 and Figure 5, respectively.

The transfer function of the perturbed system in T−Δ form can be expressed as

(28) $$\begin{array}{c} T_{MI}=-C{\left(I+\mathrm{GC}\right)}^{-1}G \end{array}$$

(29) $$\begin{array}{c} T_{MO}=-\mathrm{GC}{\left(I+\mathrm{GC}\right)}^{-1} \end{array}$$

By the small gain theorem as reported in [40], a perturbed system having uncertainty exhibits the robust stability characteristics only if the following conditions are satisfied:

(30) $$\begin{array}{c} \left|\right|T_{MI}{\left|\right|}_{\infty }<\frac{1}{\left|\right|\Delta_{l}{\left|\right|}_{\infty }} \end{array}$$

(31) $$\begin{array}{c} \left|\right|T_{MO}{\left|\right|}_{\infty }<\frac{1}{\left|\right|\Delta_{O}{\left|\right|}_{\infty }} \end{array}$$

It takes a longer period of time to evaluate the stability conditions in Equations (30) and (31). Hence, the following relations were derived to avoid the aforesaid problem:

(32) $$\left|\right|T_{\Delta }{\left|\right|}_{\infty }\;<\;1\Leftrightarrow \rho \left(M\Delta \right)<1\;\forall \;\omega \;\varepsilon \;\left[0\;\;\infty \right]$$

The stability criteria described in Equation (32) can be modified as

(33) $$\begin{array}{c} \rho (C{\left(I+\mathrm{GC}\right)}^{-1}G\Delta_{I})\;<\;1\;\forall \;\omega \;\varepsilon \;\left[0\;\;\infty \right] \end{array}$$

(34) $$\begin{array}{c} \rho (\mathrm{GC}{\left(I+\mathrm{GC}\right)}^{-1}G\Delta_{O})\;<\;1\;\forall \;\omega \;\varepsilon \;\left[0\;\;\infty \right] \end{array}$$

## 4. Results and Discussion

This section discusses the simulation results of the proposed control strategy by using Matlab/Simulink environment. Further, the tracking performance and robustness studies are conducted for uncertain parametric conditions. The following interacting tank systems are considered: (i) coupled conical tank system (CCTS) [41], (ii) coupled spherical tank system (CSTS) [29] and (iii) quadruple tank system (QTS) [42]. As presented in [43], the ideal range for gain and phase margin are between 2–5 and 30–60${}^{\circ }$ respectively. While designing the PID controller, the value of the derivative filter is taken as 100.

### 4.1. Coupled Conical Tank System

**Figure 6:**
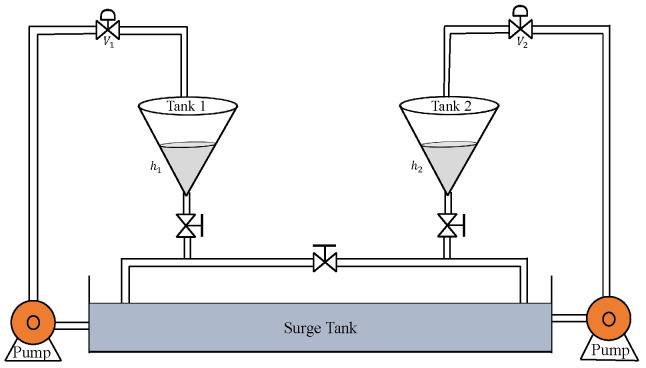
Coupled conical tank system.

The schematic structure of the CCTS is shown in Figure 6. In this, $h_{1}$ and $h_{2}$ denote the levels of tank 1 and tank 2 which need to be controlled. The manipulated variables are the input flow rates (cm^3^/s) that are controlled by control valves $V_{1}$ and $V_{2}$, respectively.

As reported in [41], the height of the tank is 50 cm, and the tank is operated around 20–25 cm by regulating the input flow rate. The TITO FOPDT process model is obtained at the operating points $h_{1}$ = 24.5 cm, $h_{2}$ = 25.6 cm. The transfer function of the CCTS is given by

(35) $$G\left(s\right)=\left[\begin{array}{cc} \frac{1.8361e^{-11.5s}}{340.7s+1} & \frac{0.723e^{-19.2s}}{415.4s+1}\\ \frac{0.74e^{-19.1s}}{407.3s+1} & \frac{1.89e^{-12.4s}}{365.6s+1} \end{array}\right]$$

The decoupler is designed from Equation (2):

(36) $$D\left(s\right)=\left[\begin{array}{cc} 0 & -\frac{\left(246.3261s+0.723\right)e^{-s}}{762.715s+1.8361}\\ -\frac{\left(270.544s+0.74\right)e^{-6.7s}}{770.91s+1.89} & 0 \end{array}\right]$$

The FOPDT model can be obtained from Equations (8)–(10):

(37) $$\begin{array}{ccc} G_{11}\left(s\right) & = & \frac{1.54}{268.99s+1}e^{-1.54s} \end{array}$$

(38) $$\begin{array}{ccc} G_{22}\left(s\right) & = & \frac{1.598}{211.9s+1}e^{-1.85s} \end{array}$$

The values of gain and phase margin are chosen as 3${}^{\circ }$ and 38${}^{\circ }$ respectively. Thus, the proposed decentralized PI controllers for the diagonal FOPDT models are obtained from Equations (18) and (19):

(39) $$\left[\begin{array}{cc} 38.83+\frac{0.151}{s} & 0\\ 0 & 27+\frac{0.123}{s} \end{array}\right]$$

Furthermore, the decentralized PID controllers are obtained from Equations (25)–(27):

(40) $$\left[\begin{array}{cc} 38.83+\frac{0.151}{s}+0.038s & 0\\ 0 & 27+\frac{0.123}{s}+0.03s \end{array}\right]$$

**Figure 7:**
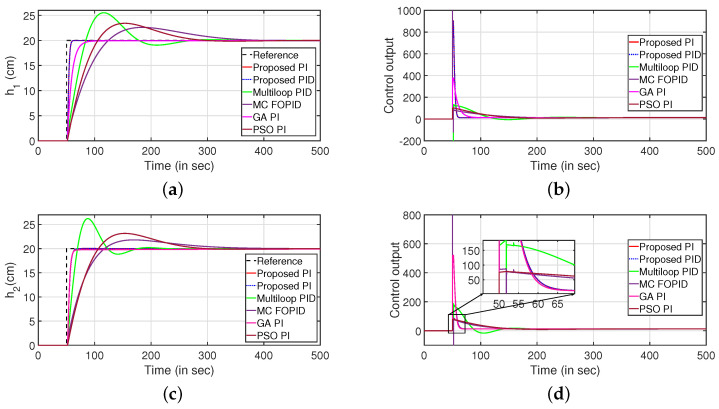
Servo response. (**a**) Level Variation in tank 1. (**b**) Control output. (**c**) Level Variation in tank 2. (**d**) Control output.

The servo response of the CCTS is presented in Figure 7. It is observed that the proposed control algorithm performs better as compared to the following methods which are multi-loop PID [23], multi-variable centralised FOPID (MCFOPID) [23], particle swarm optimization based PI [44] and genetic algorithm based PI (GAPI) [41]. The reference tracking for the variation of levels of tanks 1 and 2 are illustrated in Figure 7a,c. The corresponding controller outputs are shown by Figure 7b,d respectively. Table 1 presents the comparative analysis with other controllers by comparing the performance indices such as integral absolute error (IAE), integral of time absolute error (ITAE), and integral squared error (ISE). It is observed that the proposed controller performs better compared to other controllers.

**Figure 8:**
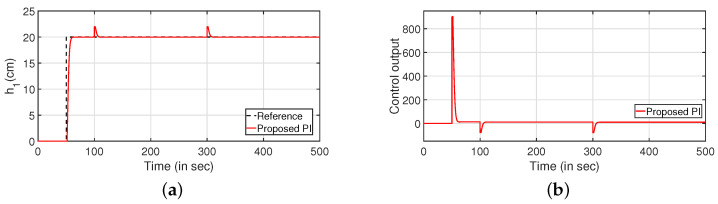

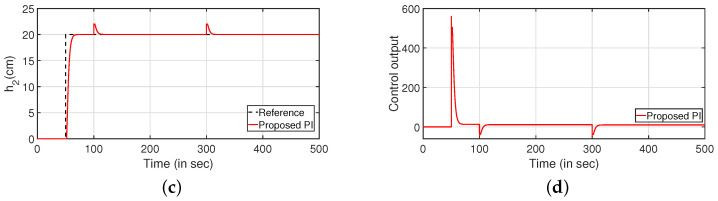
Regulatory response. (**a**) Level Variation in tank 1. (**b**) Control output. (**c**) Level Variation in tank 2. (**d**) Control output.

The regulatory response of the system is verified in Figure 8. Two different step signals are applied as input and output disturbance at 100 and 300 s, respectively. Figure 8a,c denote the reference tracking. The corresponding controller outputs are plotted in Figure 8b,d, respectively.

#### Robustness Analysis

**Figure 9:**
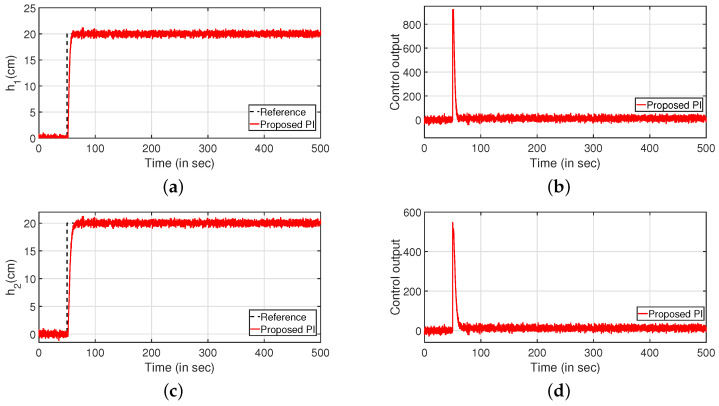
Reference tracking of PI controller with input uncertainties. (**a**) Level Variation in tank 1. (**b**) Control output. (**c**) Level Variation in tank 2. (**d**) Control output.

**Figure 10:**
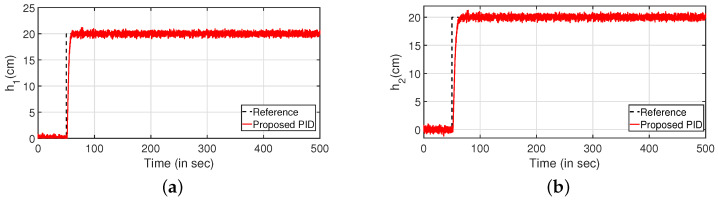
Reference tracking for PID controller with input uncertainties. (**a**) Level Variation in tank 1. (**b**) Level Variation in tank 2.

**Figure 11:**
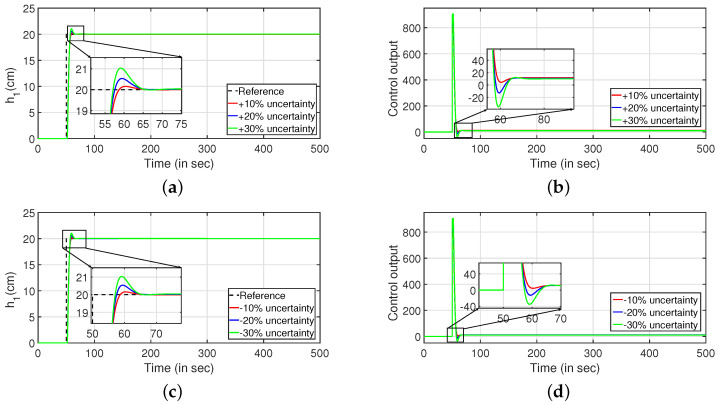
Reference tracking of Equation (37) for ±10%, ±20% and ±30% uncertainties. (**a**) Level Variation in tank 1. (**b**) Control output. (**c**) Level Variation in tank 1. (**d**) Control output.

**Figure 12:**
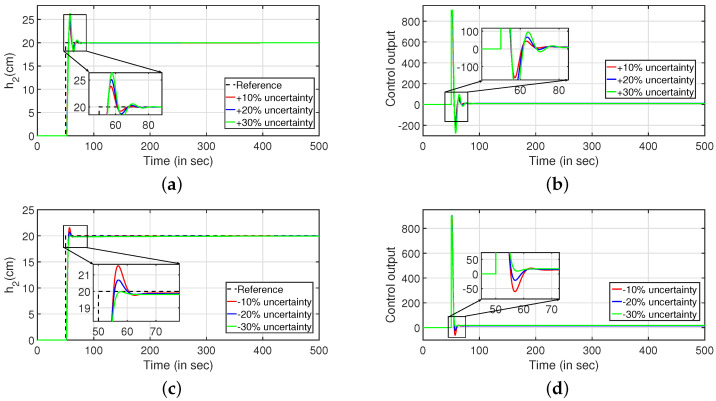
Reference tracking of Equation (38) for ±10%, ±20% and ±30% uncertainties. (**a**) Level Variation in tank 2. (**b**) Control output. (**c**) Level Variation in tank 2. (**d**) Control output.

Robustness study is analyzed by verifying the transient response of the system in presence of stochastic disturbances. Hence, white noise with power 20 is introduced into the process input as shown in Figure 9 (PI controller) and Figure 10 (PID controller). It is evident from Figure 9a,c and Figure 10a,b that the system is able to achieve the desired specifications in presence of process noise. The corresponding control outputs are illustrated in Figure 9b and Figure 9d respectively. Further, the parameters of the FOPDT model as presented in Equation (37) are changed by ±10%, ±20% and ±30% to verify the robust behavior of the proposed controller. The reference tracking in the presence of model uncertainty is shown in Figure 11. Furthermore, the response of the system when the parameters in Equation (38) are altered by ±10%, ±20% and ±30% is given by Figure 12. It is envisaged from Figure 11a,c and Figure 12a,c that the proposed control scheme is effective in tracking the desired values in presence of model uncertainties. The corresponding control outputs are given by Figure 11b,d and Figure 12b,d respectively.

### 4.2. Coupled Spherical Tank System

**Figure 13:**
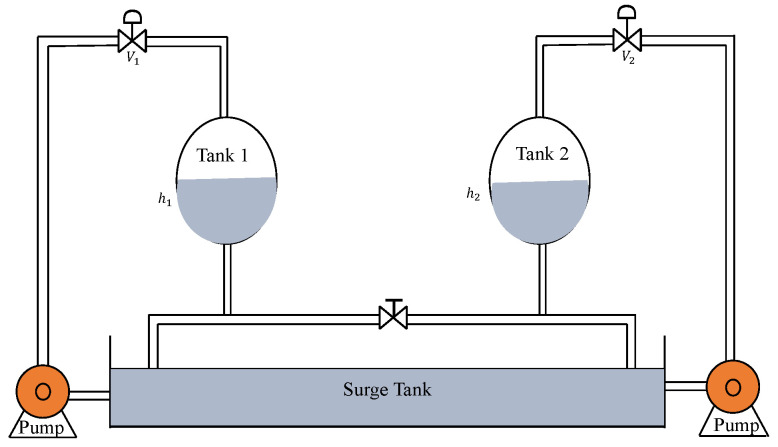
Coupled Spherical Tank System.

Figure 13 represents the schematic diagram of the CSTS where $h_{1}$ and $h_{2}$ need to be regulated by controlling the valves $V_{1}$ and $V_{2}$ respectively. Here, the input flow rate (cm^3^/s) is the manipulated variable.

As described in [29], the height of the tank is 50 cm and the corresponding operating points are $h_{1}$ = 15 cm, and $h_{2}$ = 25.6 cm. The transfer function of the CSTS is

(41) $$G\left(s\right)=\left[\begin{array}{cc} \frac{0.143e^{-0.996s}}{236.25s+1} & \frac{0.13e^{-82.305s}}{723.305s+1}\\ \frac{0.13e^{-82.305s}}{723.305s+1} & \frac{0.16e^{-0.996s}}{314.47s+1} \end{array}\right]$$

Similarly, the decouplers are designed as

(42) $$D\left(s\right)=\left[\begin{array}{cc} 0 & -\frac{\left(30.7125s+0.13\right)e^{-81.31s}}{103.43s+0.143}\\ \frac{\left(40.88s+0.13\right)e^{-81.31s}}{115.73s+0.16} & 0 \end{array}\right]$$

Further, the FOPDT model can be obtained as

(43) $$\begin{array}{ccc} G_{11}\left(s\right) & = & \frac{1.54}{268.99s+1}e^{-1.54s} \end{array}$$

(44) $$\begin{array}{ccc} G_{22}\left(s\right) & = & \frac{1.598}{211.9s+1}e^{-1.85s} \end{array}$$

The values of gain and phase margin are chosen as 3 and 45${}^{\circ }$ respectively. Thus, the proposed decentralized PI and PID controllers are obtained as

(45) $$\left[\begin{array}{cc} 22.9+\frac{1.8}{s} & 0\\ 0 & 24.25+\frac{1.5}{s} \end{array}\right]$$

(46) $$\left[\begin{array}{cc} 22.9+\frac{1.8}{s}+0.625s & 0\\ 0 & 24.25+\frac{1.5}{s}+0.375s \end{array}\right]$$

**Figure 14:**
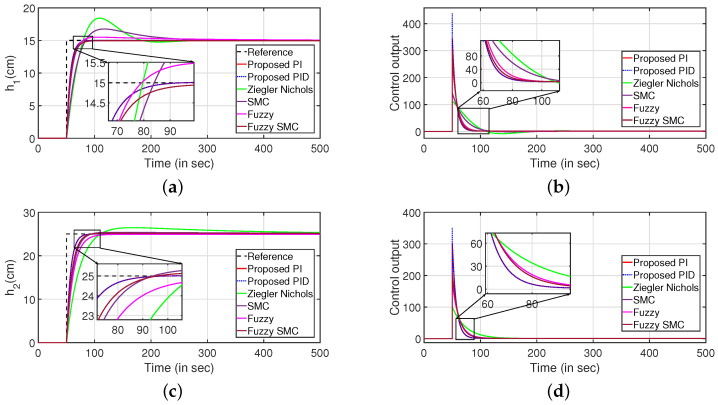
Servo response. (**a**) Level Variation in tank 1. (**b**) Control output. (**c**) Level Variation in tank 2. (**d**) Control output.

**Figure 15:**
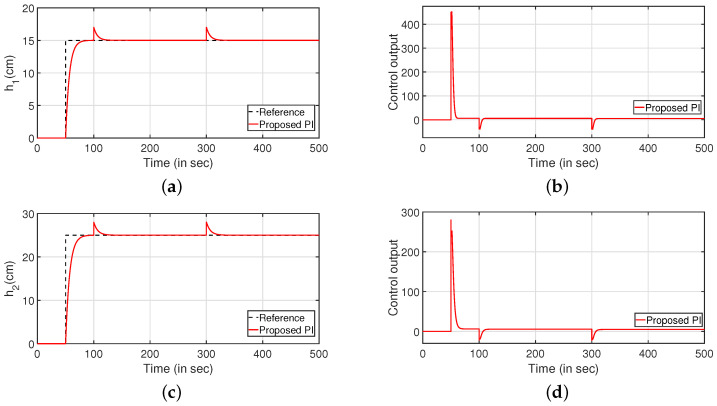
Regulatory response. (**a**) Level Variation in tank 1. (**b**) Control output. (**c**) Level Variation in tank 2. (**d**) Control output.

Simulations were performed to substantiate the effectiveness of the proposed controller for the CSTS. It can be inferred from Figure 14 that the proposed PI/PID controller exhibits better performance compared to the control algorithms as reported in [9,28,29]. Similarly, the servo response for the levels in two tanks are illustrated in Figure 14a,c respectively. Further, the controller outputs are illustrated in Figure 14b,d, respectively. Table 2 highlights the efficacy of the control algorithm. Similar to the CCTS, the regulatory response is verified by imposing disturbances in the form of step signals to input (at 100 s) and output (at 300 s) as shown in Figure 15. Figure 15a,c depict the regulatory response while the corresponding controller outputs are shown by Figure 15b,d, respectively.

#### Robustness Analysis

**Figure 16:**
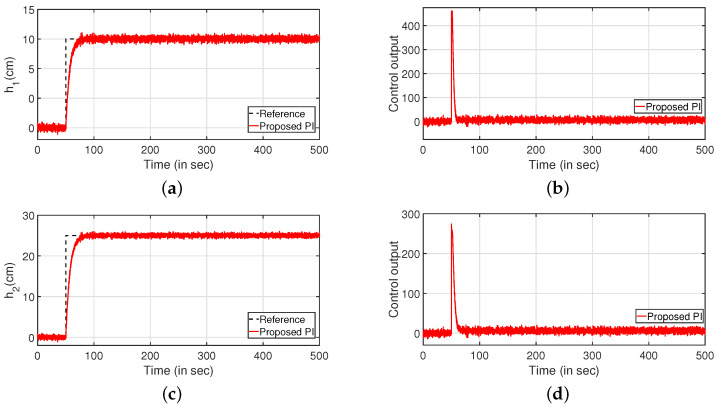
Reference tracking for PI controller with input uncertainties. (**a**) Level Variation in tank 1. (**b**) Control output. (**c**) Level Variation in tank 2. (**d**) Control output.

**Figure 17:**
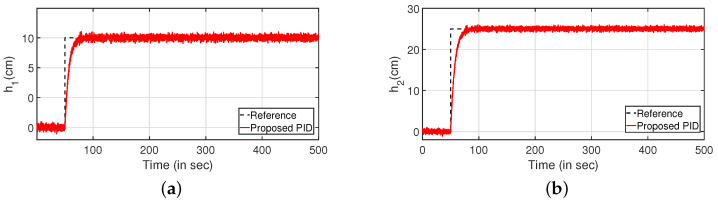
Reference tracking for PID controller with input uncertainties. (**a**) Level Variation in tank 1. (**b**) Level Variation in tank 2.

**Figure 18:**
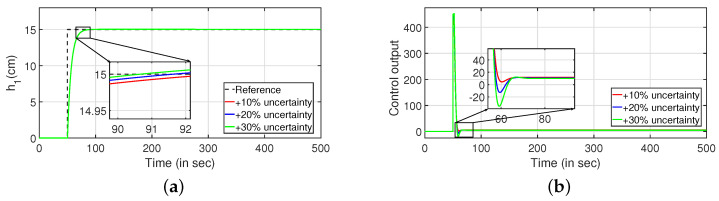

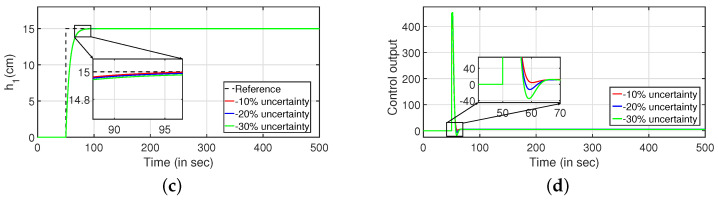
Reference tracking of Equation (43) for ±10%, ±20% and ±30% uncertainties. (**a**) Level Variation in tank 1. (**b**) Control output. (**c**) Level Variation in tank 1. (**d**) Control output.

**Figure 19:**
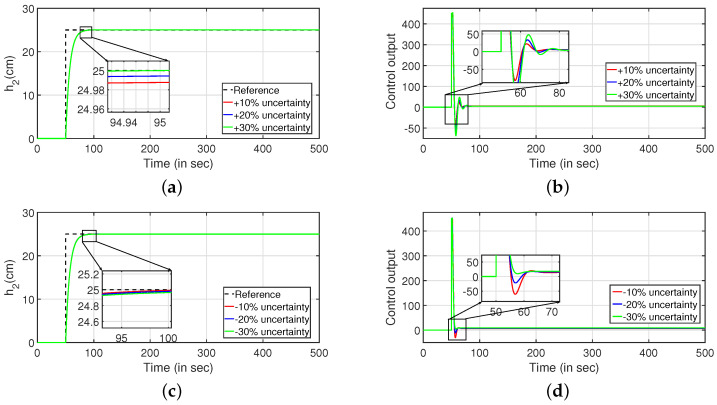
Reference tracking of Equation (44) for ±10%, ±20% and ±30% uncertainties. (**a**) Level Variation in tank 2. (**b**) Control output. (**c**) Level Variation in tank 2. (**d**) Control output.

To study the robust behavior of the closed loop system, a stochastic disturbance in the form of white noise with power 10 is applied in its input as shown in Figure 16 (PI controller) and Figure 17 (PID controller). Referring to Figure 16a,c and Figure 17a,b, it is envisaged that the system meets the desired specifications at a faster rate. The control outputs are presented in Figure 16b,d respectively. In similar approach, the robustness of the controller is achieved by changing the parameters as described in Equations (43) and (44) by ±10%, ±20% and ±30%, and it is shown in Figure 18 and Figure 19. The tracking of desired levels in presence of model uncertainties are presented in Figure 18a,c and Figure 19a,c. The corresponding controller outputs are given by Figure 18b,d and Figure 19b,d, respectively.

### 4.3. Quadruple Tank System

**Figure 20:**
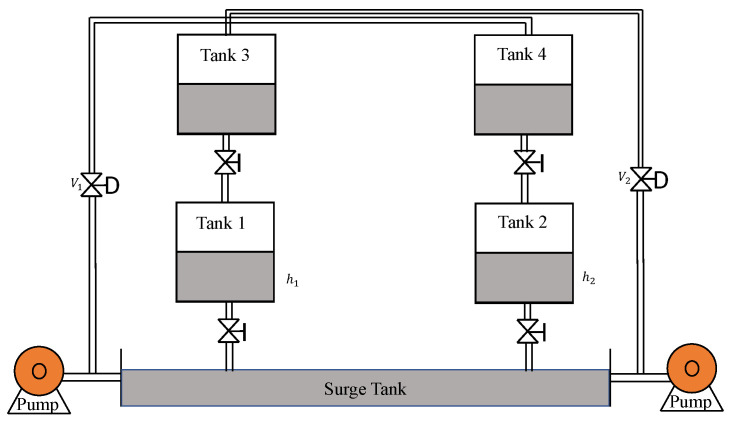
Quadruple Tank System.

The general structure of the QTS is shown in Figure 20. $h_{1}$ and $h_{2}$ denote the levels of the tank 1 and 2 which needs to be controlled by the control valves $V_{1}$ and $V_{2}$. Input flow rate (cm^3^/s) is the manipulated variable.

The transfer function of the QTS is presented in [42] and is given by

(47) $$\;G\left(s\right)=\left[\begin{array}{cc} \frac{0.834e^{-5s}}{6.57s+1} & \frac{1.39e^{-7s}}{\left(10.231s+1)(6.57s+1\right)}\\ \frac{1.271e^{-9s}}{\left(14.05s+1)(11.29s+1\right)} & \frac{0.757e^{-6s}}{11.29s+1} \end{array}\right]$$

Subsequently, the decouplers are designed as

(48) $$D\left(s\right)=\left[\begin{array}{cc} 0 & \frac{-1.667e^{-2s}}{10.231s+1}\\ \frac{-1.678e^{-3s}}{14.05s+1} & 0 \end{array}\right]$$

Furthermore, the FOPDT model can be obtained as

(49) $$\begin{array}{ccc} G_{11}\left(s\right) & = & \frac{1.54}{268.99s+1}e^{-1.54s} \end{array}$$

(50) $$\begin{array}{ccc} G_{22}\left(s\right) & = & \frac{1.598}{211.9s+1}e^{-1.85s} \end{array}$$

The values of gain and phase margin are chosen as 2 and 40${}^{\circ }$ respectively. Thus, the proposed decentralized PI and PID controllers are obtained as

(51) $$\left[\begin{array}{cc} 63+\frac{0.913}{s} & 0\\ 0 & 78+\frac{0.784}{s} \end{array}\right]$$

(52) $$\left[\begin{array}{cc} 63+\frac{0.913}{s}+0.23s & 0\\ 0 & 78+\frac{0.784}{s}+0.02s \end{array}\right]$$

**Figure 21:**
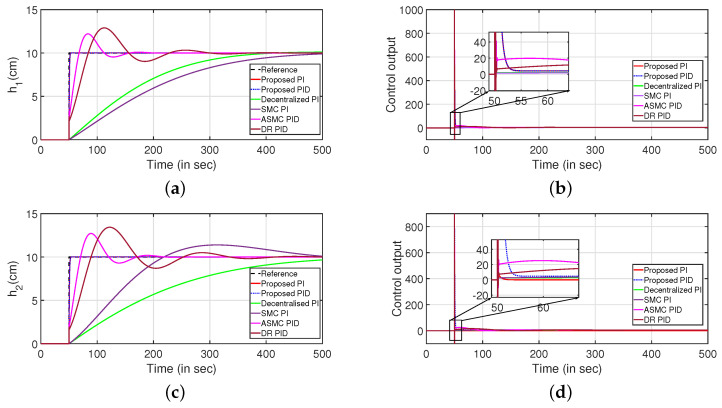
Servo response. (**a**) Level Variation in tank 1. (**b**) Control output. (**c**) Level Variation in tank 2. (**d**) Control output.

Similarly, Figure 21 indicates the servo response of the QTS. It is inferred that the closed-loop system can attain the desired reference values at a faster rate compared to the decentralized PI controller [45], sliding mode PI (SMC PI) controller [46], adaptive SMC PID (ASMC PID) controller [46], disturbance rejection PID controller (DR PID) [35]. The servo responses are shown in Figure 21a,c. The corresponding control outputs are shown in Figure 21b,d, respectively. Similarly, Table 3 indicates the performance indices of the controller that ensures the efficiency of the proposed control scheme.

**Figure 22:**
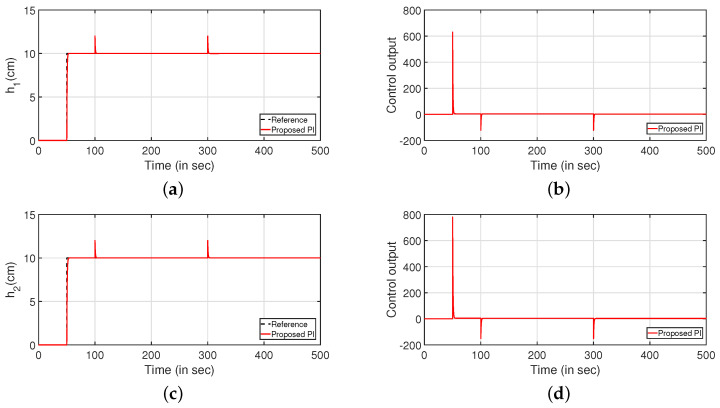
Regulatory response. (**a**) Level Variation in tank 1. (**b**) Control output. (**c**) Level Variation in tank 2. (**d**) Control output.

A similar analysis is conducted for the regulatory response Figure 22 of the system by applying two step signals as a disturbance to both input (at 100 s) and output (at 300 s) of the system. The regulatory responses of the system are presented in Figure 22a,c. The controller outputs are given by Figure 22b,d, respectively.

#### Robustness Analysis

**Figure 23:**
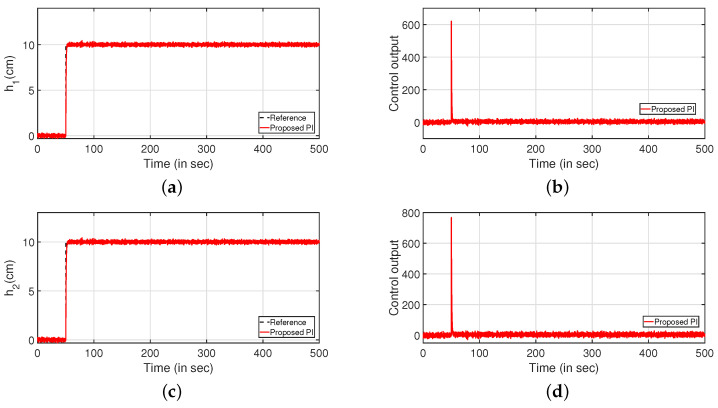
Reference tracking for PI controller with input uncertainties. (**a**) Level Variation in tank 1. (**b**) Control output. (**c**) Level Variation in tank 2. (**d**) Control output.

**Figure 24:**
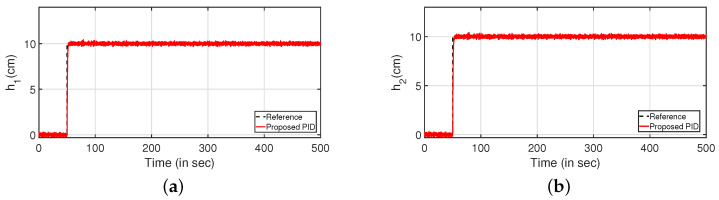
Reference tracking for PID controller with input uncertainties. (**a**) Level Variation in tank 1. (**b**) Level Variation in tank 2.

**Figure 25:**
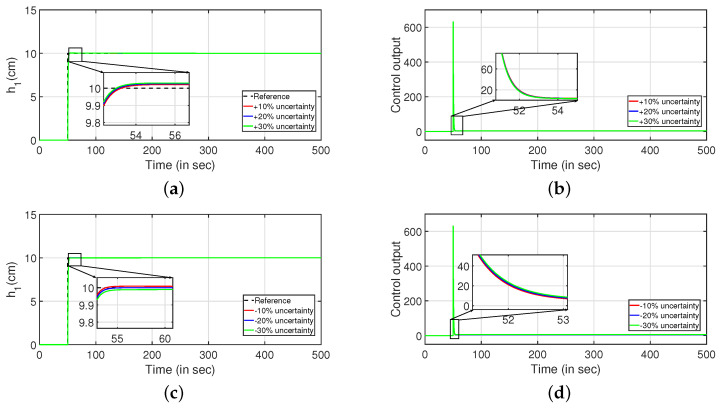
Reference tracking of Equation (49) for ±10%, ±20% and ±30% uncertainties. (**a**) Level Variation in tank 1. (**b**) Control output. (**c**) Level Variation in tank 1. (**d**) Control output.

**Figure 26:**
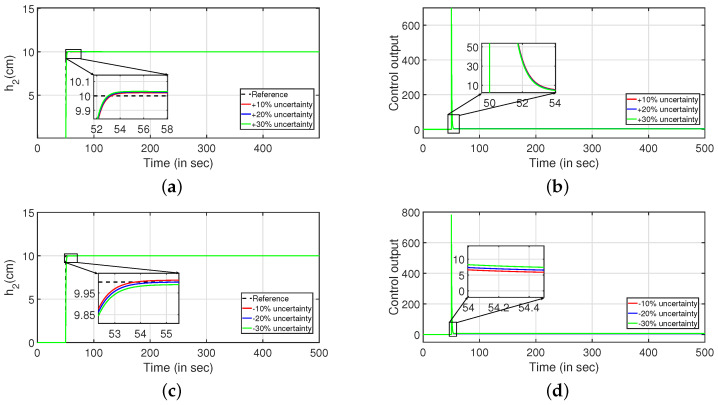
Reference tracking of Equation (49) for ±10%, ±20% and ±30% uncertainties. (**a**) Level Variation in tank 2. (**b**) Control output. (**c**) Level Variation in tank 2. (**d**) Control output.

Subsequently, the robustness analysis is carried out by applying a white noise with power 30 in the input as in Figure 23 (PI controller) and Figure 24 (PID controller). From Figure 23a,c and Figure 24a,b, it is evident that the design specifications are achieved in presence of the process noise. The controller outputs are shown by Figure 23b,d, respectively. Further, Figure 25 and Figure 26 illustrate the tracking of the system when the parameters in Equations (49) and (50) are varied by ±10%, ±20% and ±30%, respectively. The corresponding responses are presented in Figure 25a,c and Figure 26a,c while controller outputs are presented in Figure 25b,d and Figure 26b,d, respectively.

## 5. Conclusions

The paper presents a decentralized PI/PID controller based on the frequency domain specifications for various variable are coupled tank systems. The PI/PID controller parameters were derived from the specifications of gain margin and phase margin. Although gain margin and phase margin serves as the fundamentals for robustness, the main merit of the proposed controller is flexible in design aspect. To demonstrate the efficiency of the proposed controller, simulation results were performed for three different coupled tank systems. The robustness of the proposed system is exemplified by considering ±10%, ±20% and ±30% uncertainties. Furthermore, multiplicative input and output process noises are considered in the closed loop system to verify the effectiveness of the control scheme as well as output uncertainties. It is envisaged from the obtained results that the proposed controller exhibits better performance and robust behavior as compared to the aforesaid literature. 

## Figures and Tables

**Table 1:** Performance indices: coupled conical tank.

Controller	u − y	IAE	ISE	ITAE
Proposed PI	$h_{1}$	84.54	1167	5620
	$h_{2}$	109.4	1514	6426
Proposed PID	$h_{1}$	84.54	1167	5620
	$h_{2}$	109.4	1514	6426
Multiloop PID [41]	$h_{1}$	681.2	5390	7.16 × 10${}^{4}$
	$h_{2}$	431.4	3576	3.58 × 10${}^{4}$
MCFOPID [41]	$h_{1}$	917.9	7478	1.172 × 10${}^{5}$
	$h_{2}$	713.1	5893	8.228 × 10${}^{4}$
GA PI [23]	$h_{1}$	208.7	2203	1.612 × 10${}^{4}$
	$h_{2}$	173.9	1499	2.29 × 10${}^{4}$
PSO PI [44]	$h_{1}$	818.9	6815	9.513 × 10${}^{4}$
	$h_{2}$	794.1	6797	8.968 × 10${}^{4}$

**Table 2:** Performance indices: coupled spherical tank.

Controller	u − y	IAE	ISE	ITAE
Proposed PI	$h_{1}$	103.8	753.9	7087
	$h_{2}$	184.6	2248	1.23 × 10${}^{4}$
Proposed PID	$h_{1}$	103.8	753.7	7088
	$h_{2}$	184.6	2248	1.234 × 10${}^{4}$
Ziegler Nichols [29]	$h_{1}$	398.7	2268	3.799 × 10${}^{4}$
PID	$h_{2}$	789.4	6693	1.175 × 10${}^{5}$
Sliding Mode [29]	$h_{1}$	353.4	1795	3.778 × 10${}^{4}$
(SMC PID)	$h_{2}$	369.2	3335	4.531 × 10${}^{4}$
Fuzzy PID [28]	$h_{1}$	130.1	877	1.079 × 10${}^{4}$
	$h_{2}$	288.7	2960	3.052 × 10${}^{4}$
Fuzzy SMC [9]	$h_{1}$	210.5	983.3	2.713 × 10${}^{4}$
	$h_{2}$	338.3	3839	2.865 × 10${}^{4}$

**Table 3:** Performance Indices: Quadruple Tank System.

Controller	u − y	IAE	ISE	ITAE
Proposed PI	$h_{1}$	6.962	33.02	432.7
	$h_{2}$	7.311	36.63	433.4
Proposed PID	$h_{1}$	6.962	32.81	432.7
	$h_{2}$	7.311	36.5	433.5
Decentralized PI [45]	$h_{1}$	1131	6611	1.571 × 10${}^{5}$
	$h_{2}$	1559	8904	2.723 × 10${}^{5}$
SMC PI [46]	$h_{1}$	1440	8496	2.32 × 10${}^{5}$
	$h_{2}$	1021	5486	1.606 × 10${}^{5}$
ASMC PID [46]	$h_{1}$	138	420.9	1.09 × 10${}^{4}$
	$h_{2}$	192	626.4	1.67 × 10${}^{4}$
DR PID [35]	$h_{1}$	332.4	1090	3.791 × 10${}^{4}$
	$h_{2}$	452.5	1569	5.927 × 10${}^{4}$

## Nomenclature

$\Omega_{Cjj}$	Phase crossover frequency
$A_{Mjj}$	Gain margin
$\phi_{Mjj}$	Phase margin
$\omega_{Gjj}$	Gain crossover frequency
$\omega_{Pjj}$	Phase crossover frequency
$K_{Pjj}$	Proportional gain
$T_{Qjj}$	Integral time
$K_{Pjj}^{{}^{\prime }}$	Proportional gain
$T_{Fjj}^{{}^{\prime }}$	Filter time constant
$T_{Qjj}^{{}^{\prime }}$	Integral time
$T_{Djj}^{{}^{\prime }}$	Derivative time
